# Interests and Strengths in Autism, Useful but Misunderstood: A Pragmatic Case-Study

**DOI:** 10.3389/fpsyg.2020.569339

**Published:** 2020-10-06

**Authors:** Valérie Courchesne, Véronique Langlois, Pascale Gregoire, Ariane St-Denis, Lucie Bouvet, Alexia Ostrolenk, Laurent Mottron

**Affiliations:** ^1^Centre de Recherche, Centre Intégré Universitaire de Santé et de Services Sociaux du Nord-de-l’ïle-de-Montréal – CIUSSSNIM, Montreal, QC, Canada; ^2^Department of Neurology and Neurosurgery, McGill University, Montreal, QC, Canada; ^3^Department of Psychoeducation and Psychology, University of Quebec in Outaouais, Gatineau, QC, Canada; ^4^Department of Psychiatry, Child and Adolescent Sector, Centre Intégré Universitaire de Santé et de Services Sociaux du Nord-de-l’ïle-de-Montréal – CIUSSSNIM, Montreal, QC, Canada; ^5^Speech Therapy Department, Centre Hospitalier Universitaire (CHU) Sainte-Justine, Montreal, QC, Canada; ^6^Laboratoire Centre d’Études et de Recherches en Psychopathologie et Psychologie de la Santé (CERPPS), Université Toulouse – Jean Jaurès, Toulouse, France; ^7^Department of Psychiatry and Addictology, Université de Montréal, Montreal, QC, Canada

**Keywords:** autism, case-study, adolescent, strengths, interests, calendar calculation, absolute pitch, intervention

## Abstract

**Background:**

Studies on autistic strengths are often focused on what they reveal about autistic intelligence and, in some cases, exceptional and atypical reasoning abilities. An emerging research trend has demonstrated how interests and strengths often evident in autism can be harnessed in interventions to promote the well-being, adaptive, academic and professional success of autistic people. However, abilities in certain domains may be accompanied by major limitations in others, as well as psychiatric and behavioral issues, which may challenge their inclusion in support programs.

**Objectives:**

To provide an in-depth, pragmatic, real-life example of the psychological and psychiatric management of interests and strengths in an autistic adolescent.

**Method:**

An autistic teenager, C.A., with above-average calendar calculation and musical abilities, received psychiatric, neuropsychological, and language standardized and clinical assessments, combined with a measurement of his musical and calendar calculation abilities. C.A. and his parents then received psychiatric and psychological support over a 14-month period, targeting their perceptions of C.A.’s interests, strengths, and co-occurring difficulties.

**Results:**

C.A. had a verbal IQ within the intellectual disability range and a non-verbal IQ in the low mean range. Modest calendar calculation, absolute pitch, and matrix abilities coexisted with severe receptive and expressive language disorder. The discrepancy between his abilities in areas of strengths and his limitations in other domains led to anxiety, frustration, and sometimes behavioral issues. Displacing the focus from academic performance to interests, as well as promoting the use of his strengths to develop new skills independently of their short-term adaptive benefits yielded positive effects on C.A.’s self-assessment, quality of life, and behavior at follow up.

**Discussion:**

The appealing idea that abilities mostly found in autistic people, such as calendar calculation, can be directly harnessed into academic achievement and lead to paid employment may have detrimental effects, especially when such abilities are modest and associated with other limitations. These abilities should be primarily used to maximize well-being and quality of life, independently of their short-term adaptive function, which may or may not be positive.

## Background

Intense interests and special abilities in autism have been the subject of constant attention for almost a century (Feinstein, 2011). Originally only a subject of curiosity, they were quickly used as a gateway to autistic cognition, with the seminal studies of Hermelin and O’connor (i.e., 1971; 1975), Shah and Frith (1983), and Mottron and Belleville (1993, 1995). They were generally considered to have no adaptive or social utility and to be more useful to the scientists who study them than to the people who possess them. These abilities were often considered *islets of abilities* seen in so-called *idiot savants* (Feinstein, 2011), therefore not related to general intelligence, and even to this day they remain pathologized. For example, repetitive behaviors and specific interests are often considered to hinder learning in early intervention (e.g., Rogers and Dawson, 2009).

The notion of the exceptionality of abilities in autism and their uselessness as an adaptation tool both started to change at the beginning of the twenty-first century. First, a kinship between the outstanding abilities that one could find in savant autistic and non-savant autistic people was proposed (Heaton, 2009; Howlin et al., 2009; Meilleur et al., 2015). This supported the possibility that most autistic people would present an ability stronger than predicted by their apparent general level. Since then, the capacities and interests of autistics have been slowly reintroduced as valuable, at least to increase the quality of life of the people who possess them (Winter-Messiers, 2007; Chiodo et al., 2017; Davey, 2020).

Aside from this positive, but limited, trend in the scientific literature, certain aspects of this beneficial change of orientation regarding autistic capacities, associated with better acceptance of autistics as members of the human community, could have harmful side-effects for autistic people. First, employment based on skills can lead to an increase in the burden of what autistic people are exposed to in a professional environment, and therefore their anxiety to perform satisfactorily in the environment in which they work (Holmes and Annabi, 2020). Savant autistic abilities have sometimes been interpreted as suggesting that every autistic person has exceptional, innate capacities, allowing them to effortlessly learn and retain large amounts of data. This latter notion is now part of the popular image of autism, attested by television series and media stories. This unnuanced portrayal is seen as annoying by some parents (Happé, 2018). Strengths or talents, both in autism and within the neurotypical population, are to be distinguished from a gift as the latter implies that no effort or practice is needed to attain a certain level, which is not necessarily the case for a strength or a talent. Furthermore, strengths could have an adverse as well as a beneficial effect depending on context (Eigsti and Fein, 2013; Russell et al., 2019). Finally, the superiority of many autistic people in non-verbal tasks (Muth et al., 2014) and how they are underestimated by intelligence assessments (Courchesne et al., 2015, 2019) may hide that a normal distribution of intelligence is expected in autistic and non-autistic people. Uneven intelligence profiles, and general intelligence may obviously influence their learning abilities, both inside and outside their domain of expertise.

This case-study of an autistic teenager and his family focuses on the challenges arising from a direct association between the level of these strengths and expected academic performance or adaptive level. It does not question that autistic people have strengths, nor the fact that these strengths represent an advantage for themselves and the entire community.

## Case History

C.A. is the only child of a Mediterranean family who immigrated to Canada before their son’s birth. C.A.’s cousin has Tourette syndrome, but no other neurodevelopmental conditions were reported in his relatives. His mother reported having had some learning difficulties in math and sciences. She could speak English and French but her level of comprehension was limited in both, whilst C.A.’s father had a better level in both languages. French is the language used at C.A.’s school, but he was exposed to his parents’ native language, before he was exposed to French at the daycare center he attended from approximately 20 months to 4 years of age. From 4 to 5 years of age, he attended a specialized preschool in English. Motor development was delayed (sitting: 7 months, remaining upright: 12 months, walking: 20 months). After a first word at 12 months, he used only a few isolated words over a 3-year period.

C.A. was first assessed in a development clinic at 28 months of age following speech and language development concerns. Delayed onset of eye contact, apparent disinterest in other children, and hand-leading were noted. At that time, fine motor abilities were in the 12th percentile. Communication precursors were limited and speech assessment impossible due to lack of production. His adaptation to daycare routines and social demands was difficult. He also displayed an interest in vacuum cleaners, toys that made music, and cause-and-effect toys. The first combinations of two words were observed at 4 years of age. He, however, displayed early learning of the alphabet in French at around 2 years of age (for example, he compared the Mercedes logo to the letter M) and was categorizing objects by their shape at this age. The parents reported that he self-learned these skills through a cause-and-effect toy (e.g., the toy would say *Taxi* and C.A. would press the letter T). Audiology was within normal limits. Minor dysmorphic features (hypotelorism, soft and pliable ears, unilateral right clinodactyly, right single transverse palmar crease) were noted but considered to be non-clinically significant. C.A.’s first diagnosis was global developmental delay.

A second assessment took place when C.A. was 3 years 11 months old and included an ADOS (Lord et al., 2000). At that time, he produced two- to three-word non-grammatical sentences, together with stereotyped language. Toilet training had just been achieved. Eye contact was still atypical. As he was not testable by conventional tests, such as the Weschler scales, he was administered the *Eye-Hand coordination*, *Non-verbal reasoning*, and *Language* subscales of the Griffiths Mental Development Scales, normed from birth to 8 years of age. C.A’s performance on all subscales showed a significant developmental delay, of 18 months on average. For language, the delay was superior to 24 months. He received a diagnosis of autism following this assessment. No strength-oriented tests were administered at that time and hence no more precise and domain-specific conclusions can be drawn regarding his intellectual level during childhood.

As for services and interventions received, when he was 2 years old, C.A. went to a family daycare twice a week. At three he attended private sessions focused on global motricity development. At four and continuing through childhood, he received specialized services in the community and in a specialized elementary school, in addition to private services in speech and language therapy, occupational therapy, and psychology. A neuropsychological assessment conducted around age 10 yielded a heterogeneous intellectual profile (which was also found in the current study, see below) and concluded to a visuo-constructional apraxia, attention and executive functioning difficulties. Between 11 and 13 years of age, he was followed in a specialized youth mental health clinic, and re-assessed. A co-occurring mild intellectual disability and ADHD diagnosis were given. The intellectual assessment conducted at that time yielded homogeneous results (between the first and 2nd percentile, except on fluid reasoning, which was at the 8th percentile). Adaptive behaviors were in the extremely low range, which led to the diagnosis of mild intellectual disability. He was prescribed atomoxetine (10 mg) and methylphenidate (40 mg) for ADHD symptoms, with an initial positive effect on attention. He was still taking this medication during the course of this study, which began when C.A. was 13 years 8 months old. However, C.A. and his parents reported not seeing overt effect of this medication anymore. At the end of the follow-up, his family doctor prescribed a different brand of methylphenidate and a dosage of 45 mg, without additional benefit.

C.A. currently attends a specialized class for autistic youths, without other support. His parents realized just before the beginning of this study that C.A. could calculate calendar dates. Moreover, they reported that he is good at recognizing songs and has absolute pitch according to his music teacher. They contacted the research team and decided to participate in the study seeking ways to potentialize C.A.’s strengths.

## Methods

The study was initiated when C.A.’s parents sought help from the research team for optimizing their son’s calendar calculation and musical abilities. Research-oriented investigations were conducted conjointly with a multidisciplinary clinical team. Consent to use the results from the assessments and intervention for research purposes was obtained from C.A. and his parents at the beginning of the study and the Frontiers consent form for the publication of case studies was signed by the mother prior to manuscript submission. An initial psychiatric assessment session was conducted by a psychiatrist (L.M.), a psychologist (V.C.), and a psycho-educator (V.L.) with C.A. and his parents in the hospital setting at the very beginning of the study. This initial meeting was followed by an extensive cognitive, language, academic, adaptive, and psychosocial assessment, including an investigation of interests and strengths. The language assessment was conducted based on best practices for language assessment in autism (Broome et al., 2017) by a speech and language therapist (A.S-D.) while the other domains were assessed by V.C., V.L., and L.B. The assessment was conducted between 13 years 8 months and 13 years 11 months of age. Tables 1, 2 present the tests and subtests used for the cognitive, language, adaptive, and psychosocial assessment.

**TABLE 1:** Results for each test and subtest administered.

**Measured function**	**Tests or subtests used**	**Percentile**
IQ	RPM WISC-V	13 3 (FSIQ) 2 (Verbal comprehension) 3 (Visuospatial reasoning) 27 (Fluid reasoning) 8 (Working memory) 1 (Processing speed)
Expressive language	EOWPVT-4 CELF-CDN-F + WIAT-II	27 (Vocabulary) 0.2–4 (Oral expression)
Receptive language	EVIP-A CELF-CDN-F	<1 (Receptive vocabulary) 0.2 (Receptive language)
Reading	BALE+ *Le Vol du PC* WIAT-II+ *Le Vol du PC*	<0.1–2.3 (Decoding)^*a*^ 0.1–3 (Reading comprehension)
Writing	WIAT-II+BALE WIAT-II	2.3–14 (Spelling)^*b*^ 2 (Grammar and written expression)
Adaptive behaviors	VABS^*c*^	4 (Adaptive behavior composite) 4 (Communication) 7 (Daily living skills) 5 (Socialization)

*^*a*^*Decoding of regular words was within the average. Reading speed for single words was within the average. ^*b*^Spelling for regular words was within the average.*^*c*^Completed by mother.*

**TABLE 2:** Questionnaires administered and results.

**Questionnaire**	**Construct measured**	**Results**
PedsQL^TM^ 4.0	Youth’s quality of life (parent rated)	62% satisfied
	Physical	93.75%
	Emotional	45%
	Relation with peers	50%
	Studies	60%
	Youth’s quality of life (self-rated)	56.25% satisfied
	Physical	90%
	Emotional	35%
	Relation with peers	45%
	Studies	55%
FQoL	Family quality of life	
	Family interaction	4.33/5–satisfied
	Parenting	4.00/5–satisfied
	Emotional well-being	3.25/5–neutral
	Physical/Material well-being	4.20/5–satisfied
	Disability-related support	2.75/5–neutral
DASS-21	Mother’s mental health	Invalid
Parenting style and dimensions questionnaire	Self-reported parenting style	Authoritative
Parenting sense of competence	Self-reported parenting efficacy and satisfaction	High
HIBOU	Sleep issues screening	5/27 (Normal)

Three psychiatric, six individual psychotherapy, and six parental coaching sessions, as well as a mid-intervention summary session, followed the assessment sessions. These intervention sessions were conducted in order to answer the clinical needs identified during the assessment. Hence, their focus and duration were driven by clinical considerations (see below for more details about the content of the sessions). Both the father and mother were present for the initial assessment, the mid-intervention session and one of the parental coaching session. Only the mother was present for the other parental coaching sessions and the follow-up assessment. Clinicians kept detailed progress notes of each session, which were used to describe the interventions conducted. These notes were also used to document barriers, setbacks and progress observed by the clinician at each session. A final follow-up session, during which some questionnaires were re-administered, was conducted 4 months after the end of the intervention. These are preceded by an ≪^∗^≫ in the following descriptions and measure the primary (Quality of Life) and secondary (Adaptive Behaviors) outcomes targeted by the intervention.

### Cognitive, Language, and Academic Assessment (13:8–13:11 Years Old)

#### Raven’s Standard Progressive Matrices (RPM: Raven et al., 1998)

The RPM is a measure of fluid intelligence. Its administration does not require language; it is composed of five sets of 12 matrices of increasing difficulty. RPM have been shown to be suited to the assessment of autistic intelligence, especially when verbal skills are limited (Courchesne et al., 2015). It was administered to C.A. in a single session.

#### Wechsler Intelligence Scales for Children—Fifth Edition (WISC-V: Wechsler, 2014)

The WISC-V is the most widely used intelligence test. It provides information on visuospatial reasoning, fluid reasoning, verbal comprehension, working memory, and processing speed. All mandatory and supplementary subtests were administered to C.A. in two separate sessions.

#### Clinical Evaluation of Language Fundamentals—French Canadian Version (CELF-CDN-F: Wiig et al., 2009)

The CELF is a comprehensive test battery that assesses language abilities and was found to be representative of spontaneous speech in children with autism (Condouris et al., 2003). It was used here to provide information on both receptive and expressive language. It was administered to C.A. during one of the assessment sessions conducted by the speech and language therapist.

#### Wechsler Individual Achievement Test—Second Edition (WIAT-II: Wechsler, 2005)

The WIAT-II assesses academic achievements of children and adolescents. It provides information about their level in reading, written language, oral language, and mathematics. For the purpose of the present study, all subtests included in the reading and writting subscale were administered, as well as the oral expression subtest. It was also administered as part of the language evaluation and provided information regarding C.A.’s academic level in language-related subjects.

#### Expressive One-Word Picture Vocabulary Test—Fourth Edition (EOWPVT-IV: Brownell, 2000)

The EOWPVT-IV is a widely used expressive vocabulary assessment in which the participant is asked to name the pictures he is presented. This test was also part of the language assessment.

#### Échelle de Vocabulaire en Images Peabody (French Version of the Peabody Picture Vocabulary Test) (EVIP: Dunn et al., 1993)

The EVIP is the French version of the Peabody Picture Vocabulary Test, a receptive vocabulary test, in which the participant has to choose the one picture among four that best illustrates the word said by the experimenter. It was administered to C.A. by the speech and language therapist during language assessment.

#### Le Vol du PC (Boutard et al., 2006)

*Le Vol du PC* is a short story designed to assess reading speed, errors, and comprehension in youths aged from 11 to 18 years old. This was also part of the language assessment and served to assess academic level in French reading.

#### Batterie Analytique du Langage Écrit (BALE: Jacquier-Roux et al., 2010)

The BALE is a test battery assessing written language level in children from second to fifth year of elementary school (7–10 years old in a typical curriculum in Quebec). The text-reading speed and accuracy, as well as the regular/irregular and non-word reading subtests, were administered to asses reading ability. The regular/irregular and non-word spelling subtest was used to assess spelling ability. As the BALE norms are relative to the academic curriculum of each grade and C.A. was pursuing fourth grade level French in school, despite not being age appropriate, the BALE corresponded to his current level in school. It was administered to C.A. as part of the speech and language assessment.

#### ^∗^Vineland Adaptive Behavior Scales—Second Edition (VABS: Sparrow et al., 2005)

The VABS is a measure of adaptive functioning. It provides information on functioning in the following areas: communication, socialization, daily living skills, and motor skills. This test was administered to the mother during the initial assessment phase and at follow up.

## Psychosocial Assessment

### ^∗^Pediatric Quality of Life Inventory Generic Core Scales (PedsQL: Varni et al., 2001)

The PedsQL^TM^ 4.0 is a 23-item scale that measures health-related quality of life in a multidimensional manner (physical, emotional, social, and school functioning) among children and adolescents (ages 2–18) using a 5 points Likert scale going from ≪Never a Problem≫ to ≪Almost Always a Problem≫. A mean level of satisfaction for each scale is derived from averaging the scores in this domain. It is used to document outcomes in clinical trials, including with autistic youths (Sheldrick et al., 2012; Safa and Islam, 2017). The French version of the Child Self-report and the Parent Proxy-report (ages 13–18) were used in the present study for initial and follow-up assessment.

### ^∗^Beach Center Family Quality of Life Scale (Park et al., 2003), French Adaptation Directed by Chaume et al. (2019)

The Family Quality of Life Scale is a 25-item questionnaire that assesses satisfaction in five domains: family interaction, parenting, emotional well-being, physical/material well-being, and disability-related support using a 5 points Likert scale from very unsatisfied to very satisfied. It has been used with families of children with special needs (Boelsma et al., 2018) and autism (Hsiao et al., 2017). This test was also re-administered at follow-up.

### Depression, Anxiety, and Stress Scale—21 (DASS-21: Lovibond and Lovibond, 1996)

The DASS-21 is a 21-item self-reported questionnaire to measure the severity of symptoms associated with depression, anxiety, and stress in adults, which is appropriate to evaluate these symptoms in parents of autistic children (Firth and Dryer, 2013; Lai et al., 2015).

### Parenting Style and Dimensions Questionnaire (Robinson et al., 1995)

The Parenting Style and Dimension Questionnaire is a self-reported questionnaire that assesses parenting practices and categorizes them into authoritative, authoritarian, and permissive style.

### Parenting Sense of Competency Scale (Johnston and Mash, 1989), French Adaptation by Terrisse and Trudelle (1988)

The Parenting Sense of Competency Scale is a 17-item questionnaire to assess parents’ feelings about their parenting competency on a 1–6 Likert scale. Results vary from very low satisfaction to very high satisfaction. The questionnaire has been used with mothers of autistic youth (Tobing and Glenwick, 2007; Rodger et al., 2008).

### OWL-Sleep-Inventory (HIBOU: Jaworski et al., 2016)

The HIBOU is a parent-reported questionnaire to screen for sleep problems in children. It is the French Adaptation of the BEARS (Owens and Dalzell, 2005).

## Interests and Strengths

### Interests and Strengths Questionnaire for Preschoolers (ISQP)

The ISQP questionnaire (Larose et al., Submitted) was developed by experts in autism (including L.M., a co-author of this paper) and validated with autistic and typically developing children. It documents the strengths and interests of preschool-aged autistic children and their parents. It also includes questions on parental perception of the child’s strengths and interests, and documents interventions that included or targeted the child’s interests and/or strengths. It is composed of 19 multiple-choice and open-ended questions. The questionnaire was adapted and used as a parent semi-structured interview in the present study.

### Absolute Pitch Assessment

Absolute pitch was assessed through the identification of 60 musical notes (Vangenot, 2000) separated into 6 musical dictations of 10 notes. Each note lasted 1,000 ms, followed by an ISI of 2,000 ms. No feedback was provided and there was more than one octave between each consecutive note to prevent the use of relative pitch.

### Calendar Calculation Assessment

We asked C.A. to identify the weekday of 10 past dates (from year 2000 to 2018) and 10 future dates (from year 2018 to 2037), one every 2 years. For each group of dates (past and future), the questions did not involve the same month more than twice and correct answers did not fall on the same weekday more than twice.

## Intervention (14:2–15:4 Years Old)

### Psychiatric Intervention

Three psychiatric intervention sessions were conducted by a psychiatrist (P.G.) in the hospital setting with C.A. and his parents. All sessions lasted between 1 and 2 h. The first two sessions were conducted conjointly with a nurse, who acted as the contact person for the family for the psychiatric intervention. The last session was a co-intervention session with the school pedagogy specialist, with the objective of better understanding the pedagogical objectives and alternative possibilities for school programs, based on C.A.’s interests.

### Parental Coaching

Six parental coaching sessions were conducted with C.A.’s mother by a psycho-educator (V.L.). Three were conducted in the hospital setting and three at home. The father was also present in one of the session conducted at home. They all lasted between an hour and an hour and a half.

### Psychotherapy With C.A.

Six individual psychotherapy sessions (three in the hospital setting, three at home) were conducted with C.A. by a psychologist (V.C). After the first three sessions of psychotherapy and parental coaching, a mid-intervention summary session took place with the parents, C.A., clinicians (V.C. and V.L.), and a psychiatrist (L.M.) to discuss the evolution of the situation and the intervention plan for the next sessions. The last three sessions were conducted in the home setting and ended with a part conducted conjointly with the parents, C.A., and clinicians.

### Milieu Adaptation

Following the team’s recommendation and discussion with the pedagogy specialist at C.A.’s school, a meeting was held between C.A., his parents, and the school pedagogy specialist at C.A.’s school to assess his interests and needs and discuss adaptations that could be implemented.

## Follow-Up (15:8 Years Old)

A follow-up session took place 4 months after the end of the intervention. Based on the assumption that the follow-up discussion would be richer if conducted by clinicians familiar to C.A. and his parents, this session was conducted by V.L. and V.C., who respectively, conducted the parental coaching and individual psychotherapy sessions. This session included the re-administration of questionnaires assessing primary (Quality of Life) and secondary (adaptive behaviors) outcomes (see assessment section for details). An informal discussion about the family’s experience throughout the study further documented the barriers and facilitators they encountered during the intervention. The discussion was conducted in part by V.L. and V.C with C.A.’s mother alone and in part by V.C. with C.A. alone.

## Results

### Cognitive, Language, and Academic Assessment (13:8–13:11 Years Old)

C.A. presented overall intellectual functioning in the borderline range. However, his fluid intelligence assessed using the RPM was in the low average range, whereas his score on the matrix reasoning subtest of the WISC-V was in the 75th percentile, which is within the high average range for his chronological age, thus representing his better capacities. Furthermore, this high score on the Matrix subtests was drastically different from his score on the other subtest included in the Fluid Reasoning Index of the WISC-V: the Figure Weights subtest, on which C.A. obtained a score in the 5th percentile or borderline range. His score on the arithmetic subtest, assessing mental calculation abilities, was also in the borderline range around the second percentile. C.A. also presented dysprosody, depending on the topic discussed. Pronoun reversal was occasionally present. His expressive and receptive language level was significantly below that expected for his age group. His written language skills were consistent with what was observed orally. Despite relative strengths in specific domains of language, such as an expressive vocabulary within normal limits (low average range), C.A.’s language difficulties had a significant impact on his functioning and were consistent with the language ability profile of autistic youth with a co-occurring language disorder. His adaptive behavior level was in the borderline range. A higher score in motor skills is often observed in the VABS, as there is a ceiling effect for youths without a motor disorder. See Table 1 for detailed results on cognitive and language assessment.

### Psychosocial Assessment

Results from the quality of life questionnaires rated by the mother and the youth himself (see Table 2 for details) indicated good physical health (around 90% satisfaction), which is similar to normative populations, whereas the emotional, social, and school domains were lower than his physical health satisfaction (varying from 35 to 60% satisfaction) and lower than what was reported in general population studies using this questionnaire (between 78 and 84% satisfaction) (Varni et al., 2003). His mother reported being satisfied or very satisfied with almost all aspects of their family life. She reported that her husband and herself were, however, not satisfied with the support and services received for their son at school and in the community, as her family is in need of someone to help them optimize their son’s potential concerning his musical and calendar abilities. His mother rated anxiety items on the DASS-21 as ≪doesn’t apply to me, never≫, which led to the test being invalidated. Throughout the assessment the mother was reluctant to acknowledge any difficulties of challenges she was facing or to show vulnerability. Total parenting efficacy was within the mean and parental satisfaction was high. Behavioral problems were assessed using an adapted version of the tantrum questionnaire (Beauchamp-Châtel et al., 2019) to systematically document the frequency, intensity, and triggers of tantrums or meltdown. The results indicated that meltdowns occurred 1–3 times a week, for approximately 1–5 min each time, during the previous year. These meltdowns were mostly triggered when C.A. faced academic difficulties, causing him to hit his head with his palms, voice negative thoughts about himself (“loser,” “stupid,” etc.) and sometimes slap his mother (without hurting her).

### Interests and Strengths

#### Interests: Youth’s Report

C.A. reported interests in Lego^®^, videogames and YouTube videos. He mentioned that he liked listening to music but did not spontaneously mention playing music as one of his interests. He also mentioned two specific funny videos that he likes watching repeatedly on YouTube and watching documentaries about rappers.

#### Interests: Parent’s Report

History of interests included moderate or elevated interests for dinosaurs, insects, animated characters, numbers, logos, trains, dates, toys with sounds, and electronic devices during childhood. More recently, the interests reported by his parents paralleled those reported by C.A., but also included his strengths, which were not identified by C.A. as interests. Indeed, the parents reported a high or intense interest in Lego^®^, but also in calendars, music, and books (biographies), which C.A. had not mentioned. When questioned about the amount of time spent on his interests, parents reported that he plays music four times a day for 5 min each, in the context of a course for which he has to do so. He never plays music for more than 15 min on his own. He prefers to play piano by rote memory rather than by reading musical scores. In contrast, he can spend up to 60 min flipping through books and searching Google, Wikipedia, or YouTube for information on the subject he is exploring. He can also spend more than 60 min playing videogames online. He spontaneously prepared trips by gathering information on the country to be visited. The parents reported being proud of their son’s interests and press him to pursue them by encouraging him to read biographies or play music, for example, which again seem to be more related to strengths than interests.

#### Strengths: Youth’s Report

C.A. reported having strengths in calendar calculation, geography, and music recognition, and being proud of these strengths.

#### Strengths: Parental Report

C.A.’s parents reported *relative* strengths (i.e., better than his overall general level of abilities) in reproducing constructions based on a model (Lego^®^), musical memory (identifying movies with the first notes of the soundtrack), spatial orientation, electronic-device manipulation, and calculations, and an *absolute* strength (i.e., better than what most people can do) in date memory/calculation. They reported no particular strength in reading, drawing, or puzzles. The parents reported being mostly positive about their son’s strengths. They considered these as helpful to learning and not detrimental to daily activities. They also reported promoting their son’s strengths when they identify one.

#### Strengths: Clinical and Empirical Assessment

Relative or absolute strengths associated with areas of interest were clinically explored. Concerning Lego^®^ constructions, C.A. needed the help of an adult every few steps to correct errors and guide him and he was not particularly fast at completing the steps. Dates motivated him to expose himself to levels of language superior to his actual reading abilities. For example, he is interested in biographies and reported being focused on the dates. His interest in geography and politics led him to listen to the news on TV and search for information on the countries and cities he was going to visit. His apparent knowledge or understanding of politics were limited by his verbal level. For calendar calculation, C.A. was better for past dates (7 of 10 correct) than future dates (2 of 6 correct). The testing was interrupted because he expressed discomfort and anxiety when he could not provide an answer. The further away the dates were, the longer it took him to provide an answer. He reported basing his calculations on anchor dates. He remembered that movies are released on Fridays and computed his answers from the release dates of movies. He also mentioned that dates repeat every 6 years, which is not exact. His computational abilities for calendrical information was therefore in the modest range relative to other calendar calculators, but still above the average for the general population. For absolute pitch, C.A. was unable to complete the evaluation task in its original form and attempted to find the note’s name by computing explicitly its distance from an anchor note. When notes were presented one by one, C.A correctly identified 5 of 10, which is still above chance for pitch recognition.

### Psychiatric Assessment

C.A. was experiencing high levels of anxiety in his everyday life manifested by repetitive questions, sometime concurrent with behavioral issues. His anxiety comprised generalized anxiety themes (natural disasters, not having a seat in a plane) but was more often focused on pass/fail issues, such as academic success or the fear of being unable to become a financially independent adult. Although there was no indication of social anxiety, he pressured himself to succeed in school and in social interactions. Most of his repetitive questions on time schedules were related to school. Anxiety would rise quickly during any kind of assessment, when he did not know the answer, when the task difficulty increased, or when he realized he was not going as fast as his peers in an academic task. Unanswered questions, negative comments, or irritability when he did not understand something or faced an academic difficulty resulted in meltdowns.

He also reported self-depreciative thoughts, feeling discouraged about his language limitations and his learning difficulties, and voiced negative thoughts about himself (“I am a loser,” etc.). He reported academic success as being of paramount importance to succeed in life and angrily attributed his academic challenges to his autism diagnosis, which for him encompasses all his challenges. His outbursts, which started when he was around 12 years old, circularly increased his anxiety and low self-esteem. He could calm down rapidly when his parents were able to reassure him and remind him of his strengths. He expressed feelings of shame and guilt for not being able to control them. Agreeing with him to alleviate this aspect of his anxiety resulted in more collaboration. He explicitly identified his best moments as non-school periods, holidays, and travel.

### Intervention (14:2–15:4 Years Old)

The general goal of the intervention was to improve personal and family quality of life and improve C.A.’s general adaptation including, but not limited to, adaptive behaviors. The effects of the intervention were assessed at follow-up, 4 months after the end of intervention. The follow-up session therefore included administration of quality of life and adaptive behaviors questionnaires, but also focused on discussing the general well-being of C.A. and his parents.

#### Psychiatric Intervention

For C.A., the main axes of the intervention were the validation of his emotions and needs, re-explaining the school classification process, psychoeducation about the challenges associated with autistic signs vs. those associated. e.g., with a language disorder, and cognitive restructuring of the beliefs about how one can contribute to society and live a fulfilling life. For his parents, the intervention focused on promoting independence at home, encouraging them to limit their answers to C.A.’s repetitive questions (i.e., teaching them to reformulate their answers in several distinct terms, with the use of a visual support when possible). We also highlighted positively reinforcing good behavioral management, which they were able to sustain despite periods of increased school-induced stress or increased behavioral problems.

#### Parental Coaching

The focus of these sessions was to help C.A.’s parents in seeking learning opportunities that were suited for him, for example because they do not rely on language. They were encouraged to organize activities around his interests and strengths, regardless of their level or potential effect on their son’s future. The sessions were oriented toward the acceptance of their son’s limitations. The importance of pursuing pleasant activities, with no learning goal *per se*, was highlighted. Sessions also included stress and emotional regulation tools to help C.A.’s mother deal with her own anxiety and provide her with tools (emotional validation, use of the thermometer metaphor, breathing exercises) to better react to her son’s anxiety and emotional outbursts. These sessions validated C.A.’s parents’ efforts and devotion. Despite being reluctant to acknowledge the difficulties she was facing and the emotions accompanying these challenges, C.A.’s mother was deeply touched when validated in her parenting practices or in the emotions she could be facing.

#### Psychotherapy With C.A.

The goals of these sessions paralleled those of the parental coaching sessions. We provided C.A. with emotional regulation tools (i.e., deep breathing and visually tracking his stress level on a scale). Psychotherapy also focused on the understanding and acceptation of his limitations. We encouraged him to develop more autonomy so as to experience success in various domains and not just focus on academic achievement. He was also oriented to harness his strengths and pursue his interests through playful and pleasant activities with the psychologist (i.e., building with Lego, playing music, discussing movies, etc.).

#### Milieu Adaptation

For his current school year, C.A. was oriented toward a program to learn semiskilled trade jobs. One was to wash dishes and he reported hating it. He repeatedly said he wanted to pursue his academic education and learn new academic skills. He was first provided with self-taught didactic material to fulfill his interest in learning academic subjects. In parallel, the team coordinated with the school pedagogy specialist to re-orient him toward a program focused on academic subjects. At the end of the follow-up he had recently been moved to a different class and program in which more academic work was performed, but in which he still pursued work placement in manual jobs. This program change was deemed necessary so that C.A.’s school program would consider C.A.’s *interest* in school and academic subjects, regardless of his *level* in this domain.

### Follow Up (15:8 Years Old)

All aspects of C.A.’s self-reported quality of life (physical, emotional, relationships, and studies), despite still being lower than population norms, showed increases at the follow-up assessment (see Table 3). During the follow-up discussion C.A. reported being happier in his new class, in which most of his friends from the previous year also were. He also reported liking the most recent job placement he had, disassembling electronics. He is now aware that completing a regular degree in college or university is not a realistic objective, but still has not figured out what type of occupation he would like to have after high school or if he would like to pursue his education in adapted programs. He was proud to succeed in doing some tasks that he previously thought he could not do by himself, such as cooking simple meals, and was working toward becoming more independent. He indeed wants to be able to live independently as an adult, but is not motivated to help around the house for now, as he is still young. He feels depressed about his communication difficulties and sees this as the main challenge in his acceptance of autism. He would like to have a girlfriend but is afraid his communication challenges will be a barrier to this aspiration. Overall, C.A. now has more realistic expectations concerning his strengths and he better understands his limitations. His self-assessment is therefore more accurate and he has an acute understanding of how he is regarded by others and how his own future could be challenging, which is painful for him. His mother perceives this as a loss of motivation and hope and deplores this change. However, the family quality of life reported by C.A.’s mother has shown improvements, as has the self-reported quality of life.

**TABLE 3:** Pre- and Post-intervention scores for primary and secondary outcomes.

**Questionnaire**	**Construct measured**	**Pre–Post scores**
FQoL	Family quality of life (/5)	3.70–4.60
	Family interaction	4.33–4.67/5
	Parenting	4.00–4.5/5
	Emotional well-being	3.25–4.75/5
	Physical/Material well-being	4.20–4.6/5
	Disability-related support	2.75–4.5/5
PedsQL^TM^ 4.0 self-rated	Youth’s quality of life (% satisfaction)	56.25–66.5%
	Physical	90–91%
	Emotional	35–50%
	Relation with peers	45–60%
	Studies	55–65%
VABS	Adaptive behaviors (percentile)	4th–3th
	Communication	4th–2th
	Daily living skills	7th–4th
	Socialization	5th–6th

According to the parental reports, the repetitive questions about his school schedule were at a tolerable level and the change of class was truly helpful for both C.A. and his parents. During an informal discussion about the study, C.A.’s mother stated that she appreciated having had a space to talk. She realized how important it is to emphasize things other than school and to enhance pleasant activities in her son’s life. She stated examples of how she is now trying to promote his autonomy by asking him to help around the house with various tasks, although unsuccessfully. On a more negative side, C.A.’s measured adaptive behaviors had not improved. C.A.’s mother remained convinced that her son’s abilities have been “given” to him for some purpose, and was unsatisfied by the intervention in this regard.

## Discussion

### Summary of Findings

We presented here the case report of an autistic adolescent with modest abilities in calendar calculation and musical memory, adaptive behavior in the mild to moderate disability level, uneven task-dependent non-verbal IQ, and verbal abilities in the severe disability range. Interests, cognitive, psychiatric, and adaptive measures are reported, as well as psychoeducational, psychological, and psychiatric interventions and their short-term consequences. Illustrated by this case-study, we will now discuss the relation between relative and absolute strengths and general intelligence, and the positive and negative effects of the expectations grounded on them.

### Interests, Strengths, and General Level of Intelligence

C.A’s uneven profile is characterized, as is the case for many autistic people, by an important discrepancy between fluid intelligence and verbal abilities. He also presents domain-specific performances discrepancies: some areas (dates) and operations (calendar calculation) are performed at a much higher level than others (arithmetic). Therefore, intelligence cannot be deduced from his verbal and adaptive abilities, and is task-dependent. The measurement and practical use of his fluid intelligence are bounded to specific operations and materials, at least at time of assessment. His measured fluid intelligence is in the low average range in one test, but a peak in matrix reasoning on another one indicates that it could be underestimated (see below for more details). How this profile can be modified by access to new materials and education in general remains an open question, but this profile can be considered as characteristic of autism. Given that the level attained in the domain of expertise tends to increase with age and intelligence, strengths (both relative and absolute), despite being intrinsic to the autism diagnosis, may necessitate practice. The level attained can be experience- and intelligence-dependent.

Strengths in autism can be observed in individuals with superior intelligence, but also in individuals with lower intellectual potential. Peaks of abilities and general intelligence are not properly reflected by the concept of “islets of abilities” disconnecting them from general intelligence, and there is indeed a link between the level attained in the ability and *g factor* (general intelligence) (Hermelin and O’connor, 1986; O’Connor and Hermelin, 1988). C.A.’s abilities are among those that may reach an exceptional level within an autistic presentation. Although they exceed what most non-autistic and autistic people can do, they are of a modest level relative to other published savant abilities in the same domains (Mottron et al., 1999, 2006; Thioux et al., 2006; Bouvet et al., 2019). Therefore, the expectations for translating these strengths into direct and immediate adaptive outcome need to consider the context of domain/task-specificity but also the correctly measured general level of intelligence of the person.

The recent tendency to use interests and strengths to promote learning in autism should therefore be enriched by distinguishing between intense interests (being good at vs. being interested in), strengths (absolute, relative), intelligence and transferability to other domains. Recommendations to use interests were initially limited to using them as external reinforcements (e.g., Charlop-Christy and Haymes, 1998). More recent and rare recommendations suggest using the area of interest as learning material (Baker, 2000; Winter-Messiers et al., 2007; Courchesne et al., 2016; Ostrolenk et al., 2017). As for strengths in autism, they have long been included in interventions, with principles such as using visual support (Mesibov and Shea, 2010). This case-report suggests that the use of interests in intervention should be individualized as a function of the person’s relative and absolute strengths, and transferability of abilities from the strong domain to other domains is not necessarily straightforward or doable at all. Following Dawson et al. (2008), these interests should at least lead to making relevant material available to the person, and critically observing what happens next.

### The Positive and Negative Effects of Expectations Based on Strengths

C.A. has anxious and depressive manifestations centered on academic success and his future as an independent adult, which leads to mild behavioral problems. His parents also reported preoccupations regarding their son’s future, hoping that their son can find a way to use his abilities to learn academic skills. The pressure C.A. puts on himself to achieve better academic performance, in addition to the familial and institutional pressure to see immediate effects of his strengths on other areas of learning, contribute to generating anxiety. This results in intense frustration and behavioral problems when facing difficulties, damaging to his self-esteem and family life. It also contributes to the task-dependence of his cognitive performance, as this could explain the discrepancy found between the two versions of the matrix tests that he was administered (13th vs. 75th percentile). In the RPM (13th percentile), the difficulty, and therefore failures, increase within each of the five sets, in addition to increasing between the sets, and all items are administered regardless of the number of errors. In the Wechsler version (75th percentile), the increase in difficulty is constant and the test is stopped after three consecutive errors, which minimizes the total number of errors. Furthermore, throughout testing, a decrease in anxiety was observed when the administrator intervened, for instance by repeating that it was normal not to know all the answers. Hence, the context and C.A.’s state of mind may directly influence his ability to perform.

Interventions focused on helping him with his anxiety, expectations, and emotional regulation were conducted over a 14-month period with his parents, his school, and C.A. himself. These interventions attempted to shift the focus from academic success to academic learning, both at school and within the family. This focus did not fully correspond to the family’s expectations and led to some frustration that the mother expressed to the team. Despite the fact that both C.A. and his parents were in search of a silver lining for their son’s calendar calculation ability, to which the intervention did not answer, an improvement in C.A.’s well-being and behavior was seen at the 4-month follow-up and at the end of the intervention. However, we cannot be certain that the measured improvements will last, or that they were due to the intervention.

### Adaptive Outcome vs. Quality of Life Associated With Interests and Strengths

C.A. is interested in learning and accumulating information about themes such as geography, movies, biographies, and dates, and has above average calendar calculation and absolute pitch abilities. Our observations indicate a potential risk of assuming a direct link between interests and strengths, and academic potential. This shortcut contributes to increasing the expectations for better academic performance, in turn increasing his anxiety and feelings of being a failure. Although C.A.’s interests and strengths are related to academic subjects, they do not necessarily lead to increased immediate academic performance. In contrast, C.A.’s interests and strengths greatly contribute to his well-being and self-esteem as a teenager. He connects with his peer and family through his interest in videogames, rap videos, movies, and travel. His strength in music allows him to play in a marching band and perform publicly, which is rewarding for him both socially and because it makes his parents proud, while his calendar calculation abilities impresses others and makes him feel unique. These interests and strengths could therefore further translate into skills or knowledge increasing his quality of life, even if it may not directly translate into better adaptive behaviors or employment (Winter-Messiers, 2007; Davey, 2020).

### What Can We Learn From This Case Study?

How can one harness the potential positive effect of interests and relative and absolute strengths? Interests and strengths can contribute to quality of life. They are associated with the experience of positive emotions (Sasson et al., 2012) and can positively affect self-esteem (Chiodo et al., 2017; Happé, 2018) even without an explicit attempt of increasing knowledge of related skills or being a pathway to employment. For example, mood and intrinsic motivation is enhanced when autistic people approach their area of interest (Sasson et al., 2012) and this could be useful for interventions (Davey, 2020). Concerning strengths, their level is independent from their direct usefulness for adaptive outcomes, such as academic performance or paid employment. Promoting the inclusion of interests at home and at school should therefore be cautiously distinguished from expectations regarding the level attained in those domains of interests.

The domains of relative and/or absolute strengths, as well as the domains of deficits, can also be useful for intervention or education by informing which tools or teaching methods are more or less suited for the individual. They can be informative as to what domain or type of material is particularly suited to promote learning for the person. Such strengths may or may not immediately, or ever, lead to a better adaptive outcome, but may contribute to acquiring new skills, hence enhancing general adaptation by benefiting self-esteem, well-being, and mental health. For C.A., being able to pursue his academic curriculum at his own pace, despite the fact that this might not lead to his obtention of a high school diploma, corresponds to his interest and contributes to his self-esteem and feeling of inclusion in society, as he is doing something similar to his peers. Hence, pursuing his education in an adapted college program or finding customized employment which would build on his strengths, could be promising avenues for promoting well-being and quality of life in general.

In conclusion, interests and strengths in autism may not directly lead to an instrumental outcome, such as obtaining paid employment, despite their personally rewarding value and societal utility. For example, the person who has written the most validated Wikipedia articles is autistic^1^. Thus, provided that he is given access to opportunities and becomes an empowered citizen, the possibilities are endless for 16 year-old C.A. to live a fulfilling life and contribute to society through a paid job or not.

### Limitations

This case study has several limitations. We did not have access to the test protocols for the assessments C.A. completed prior to his participation in this study. Such information would have allowed us to better characterize his early cognitive profile and how his strengths and weaknesses evolved with time. More information regarding language development in the three languages he was exposed to would also have been useful to better characterize how this impacted his development in general and hyperlexia in particular, as it was shown that bilingualism can have positive impact on certain cognitive tasks (Trelles and Castro, 2019). Further, no strength-oriented assessment was conducted during preschool or school age. A valid assessment of non-verbal abilities using tests such as the Raven’s Progressive Matrices or a formal evaluation of hyperlexia would have been useful to document these early strengths. We were hence limited to infer those strengths from parental reports and qualitative information included in previous reports.

Second, the assessment conducted in the present study was also incomplete in that it did not include many mathematic subtests, nor many music tests. It is possible that some additional strengths in those areas would have become apparent through further assessments, although these turned out to be very challenging and distressing for C.A.. The assessment did not include a direct assessment of distress for C.A. (anxiety or depression scale), which prevented us from doing a pre-post comparison on such measures. It was also relatively short term (14 months).

Third, the measures and intervention mostly relied on C.A.’s mother, as she was more available and hence participated in all assessment and intervention sessions, while the father only attended a few. Finally, it is limited by its design as this is a single case study used to illustrate and discuss how interests and strengths in autism can be harnessed to avoid potential detrimental effects to well-being and quality of life. More examples of both beneficial and detrimental effects of using interests and strengths in intervention in autism are needed.

## Ethics Statement

The studies involving human participants were reviewed and approved by the CER CIUSSS-NIM. Written informed consent was obtained from the individual(s) legal guardian/next of kin for the publication of the present case-study including anonymized images or data.

## Author Contributions

VC and LM contributed to the study design, data collection and analysis, intervention with the family, and writing and revisions of the manuscript. VL contributed to the study design, data collection and analysis, intervention with the family, and revisions of the manuscript. PG contributed to the data collection and analysis, intervention with the family, and revisions of the manuscript. AS-D contributed to data collection and analysis and revisions of the manuscript. LB contributed to the study design, to the data collection and analysis, and to the manuscript revisions. AO contributed to the study design and manuscript revision. All authors contributed to the article and approved the submitted version.

## Conflict of Interest

The authors declare that the research was conducted in the absence of any commercial or financial relationships that could be construed as a potential conflict of interest.

## Footnotes

1 https://xtools.wmflabs.org/pages/fr.wikipedia.org/Tsaag%20Valren
